# Safety, pharmacokinetics, and pharmacodynamic properties of oral DEBIO1143 (AT-406) in patients with advanced cancer: results of a first-in-man study

**DOI:** 10.1007/s00280-015-2709-8

**Published:** 2015-02-27

**Authors:** Herbert I. Hurwitz, David C. Smith, Henry C. Pitot, Jeffrey M. Brill, Rashmi Chugh, Elisabeth Rouits, Joseph Rubin, John Strickler, Gregoire Vuagniaux, J. Mel Sorensen, Claudio Zanna

**Affiliations:** 1 Department of Medicine, Duke University School of Medicine, DUMC 3052, Durham, NC 27710 USA; 2 University of Michigan, Ann Arbor, MI USA; 3 Mayo Clinic, Rochester, MN USA; 4 Ascenta Therapeutics, Malvern, PA USA; 5 Debiopharm International SA, Lausanne, Switzerland

**Keywords:** IAP, Apoptosis, AT-406, DEBIO1143, Cancer, Resistance

## Abstract

**Purpose:**

To assess safety/tolerability, pharmacokinetics (PK), pharmacodynamics (PD), and antitumor activity of DEBIO1143, an antagonist of inhibitor apoptosis proteins.

**Methods:**

This first-in-man study in patients with advanced cancer used an accelerated dose titration design. DEBIO1143 was given orally once daily on days 1–5 every 2 or 3 weeks until disease progressed or patients dropped out. The starting dose of 5 mg was escalated by 100 % in single patients until related grade 2 toxicity occurred. This triggered expansion to cohorts of three and subsequently six patients and reduction in dose increments to 50 %. Maximum tolerated dose (MTD) was exceeded when any two patients within the same cohort experienced dose-limiting toxicity (DLT). On days 1 and 5, PK and PD samples were taken.

**Results:**

Thirty-one patients received doses from 5 to 900 mg. Only one DLT was reported at 180 mg. No MTD was found. Most common adverse drug reactions were fatigue (26 %), nausea (23 %), and vomiting (13 %). Average *t*_max_ and *T*_1/2_ was about 1 and 6 h, respectively. Exposure increased proportionally with doses from 80 to 900 mg, without accumulation over 5 days. Plasma CCL2 increased at 3–6 h postdose and epithelial apoptosis marker M30 on day 5; cIAP-1 levels in PBMCs decreased at all doses >80 mg. Five patients (17 %) had stable disease as the best treatment response.

**Conclusion:**

DEBIO1143 was well tolerated at doses up to 900 mg and elicited PD effects at doses greater 80 mg. Limited antitumor activity may suggest development rather as adjunct treatment.

**Electronic supplementary material:**

The online version of this article (doi:10.1007/s00280-015-2709-8) contains supplementary material, which is available to authorized users.

## Introduction

Inhibitors of apoptosis proteins (IAPs) may play a role in the development of cancer [1, 2]. Their over-expression has been linked not only to tumor growth and poor prognosis, but also to low treatment response or resistance [2, 4]. Therefore, the IAP protein family is generally considered a promising target for cancer drug development [2, 5, 6]. So far, six IAP antagonists have entered clinical development [3]. One of these is DEBIO1143 (formerly AT-406, SM-406), a small molecule mimetic of second mitochondria-derived activator of caspase (SMAC) [7].

In vitro studies have demonstrated DEBIO1143 to inhibit cell growth in various human cancer cell lines [2, 4] through binding of X-chromosome-linked IAP (XIAP) and cellular IAPs 1 and 2 (cIAP-1 and -2). DEBIO1143 rapidly induced degradation of cIAP-1 in a cell-free functional assay [7] and apoptosis in xenograft tumors. Moreover, it was able to enhance the antitumoral effects of irradiation or various chemotherapeutic agents in multiple mouse cancer models [1, 4, 8]. Preclinical data further revealed good oral bioavailability in mice, rats, dogs, and non-human primates, enabling PK/PD modeling to predict tumor and plasma concentrations in humans [1].

Multiple high doses (40–120 mg/kg/day) induced hepatotoxicity in rats. In dogs, liver cell degeneration was seen at 3 and 10 mg/kg/day. In 4-week toxicology studies, the severely toxic dose (STD) in the rat was determined at 40 mg/kg and the highest non-severely toxic dose (HNSTD) in a non-rodent species at 1 mg/kg in dogs. Based on metabolism data and observed adverse events (AEs), the dog was considered the most relevant species, in line with reports on other IAP inhibitors [9]. The no observable adverse event level (NOAEL) of 1 mg/kg in dogs led to a calculated starting dose of 5 mg in humans. An intermittent dosing schedule was chosen to further mitigate the risk of unacceptable toxicity when entering clinical development.

The primary objective of this first-in-man study was to characterize the safety and determine the maximum tolerated dose (MTD) and schedule of DEBIO1143 when administered to patients with advanced solid tumors and lymphomas. Secondary objectives were to explore (a) PK of DEBIO1143, (b) any PD effects, (c) any observable antitumor activity during the trial, and (d) its correlation with PK.

## Materials and methods

### Design

This was a multicenter, uncontrolled, open-label, dose-escalation study on DEBIO1143 in patients with advanced cancer. It employed an accelerated titration design for dose escalation with 100 % dose increments in consecutively enrolled single patients until drug-related grade-2 toxicity was observed during the initial treatment cycle (until day 28 or day 21 as per protocol amendment). If this was the case, cohort size was expanded to three patients and dosing increment was reduced to 50 % of the last dose. Dose escalation was to be stopped at the MTD which was considered exceeded if at any dose level more than one patient experienced dose-limiting toxicity (DLT) during the first treatment cycle. DLT was defined as any of the following: (a) non-hematological toxicity of grade ≥3 (excluding nausea, vomiting, diarrhea unless not controlled by maximal antiemetic/diarrheal therapy for >24 h); (b) anemia or neutropenia of grade ≥3 or thrombocytopenia of grade 4 or any grade if associated with clinically significant bleeding; (c) any AE resulting in dose delay or reduction; (d) any toxicity considered dose-limiting by the investigator. If only one out of the three patients of a cohort experienced drug-related DLT, the cohort was expanded by another three patients to be treated at the same dose level. If none of these additional patients experienced DLT, the dose escalation by 50 %, rounded down to the nearest capsule strength combination, continued in the next cohort of three patients.

Pharmacokinetic samples were taken from all patients on day 1 (predose, 0.5, 1, 2, 3, 4, 6, 8, 12, 18 h postdose) and on day 5 (predose, 0.5, 1, 2, 3, 4, 6, 8 h postdose) of the first cycle. In addition, for exploratory PD analysis of IAP inhibition and activation of apoptosis, optional skin and tumor biopsies were taken from consenting patients on days 1 (predose) and 5 and blood samples on day 1 (predose, 1, 3, 6, 8, 12 h postdose), day 2 (predose), and day 5 (3 h postdose).

The study was compliant with all applicable legal obligations, the requirements of the Declaration of Helsinki and Good Clinical Practice. It was approved by the institutional review boards of the three participating sites and registered under Clinicaltrials.gov (identifier: NCT01078649).

### Patient population

Eligible were male and female adult outpatients with histologically confirmed advanced or metastatic solid tumors or lymphoma for which no life prolonging or appropriate standard therapy was available. Patients had to be ambulatory (Eastern Cooperative Oncology Group (ECOG) performance status ≤1) with adequate hematological (ANC ≥1,500/mm^3^; hemoglobin >9.0 g/dL; platelet count ≥100,000/mm^3^), renal (creatinine ≤1.0 × upper limit of normal (ULN) or creatinine clearance of >60 ml/min), hepatic (serum albumin ≥3.0 gm/dL; total bilirubin <1.0 × ULN; aminotransferases and alkaline phosphatase ≤2.5 × ULN, including negative hepatitis testing), and cardiac function without evidence of QTc prolongation.

As clinically significant bleeding formed part of the definition of DLT, further exclusion criteria were a history of gastrointestinal bleeding during the preceding year, of treatment-requiring diabetes mellitus, or any condition associated with chronic inflammation (e.g., rheumatoid arthritis, inflammatory bowel disease, chronic infections) or affecting copper accumulation or regulation (e.g., Wilson’s disease).

Last radiation and intake of steroids had to date back at least 14 days from study entry (thoracic radiation 28 days); patients had to be clinically stable and to have recovered to toxicity grade ≤1 from any prior cancer therapy. Patients had to have never received IAP inhibitors before. All patients had to give written informed consent to be enrolled in the trial.

### Treatment

Oral treatment had to be taken daily on days 1–5, initially every 14 days, later every 21 days as per protocol amendment. The amendment was put in place to be more conducive to future combination with common chemotherapy regimens and to reduce the potential for adverse drug reactions (ADRs) through an additional week of recovery between doses. The starting dose of 5 mg was increased in subsequent cohorts, based on DLT observed by the end of cycle 1. Stable or responding patients who experienced DLT were allowed to continue therapy at the next lower dose, once those had resolved to grade ≤1 within 2 weeks. End of treatment was triggered by disease progression, unacceptable toxicity, or withdrawal from the study for any reason. Cancer therapy other than DEBIO1143 was not allowed, but supportive care measures were. Concomitant treatment with aspirin at doses >81 mg/day or with any anticoagulants was prohibited.

### Endpoints

#### Safety

The incidence of AE, ADR, and DLT was recorded at all scheduled visits (on days 1, 15, and 28 of each cycle and additionally on days 5, 8, and 22 of cycle 1) and graded according to the Common Terminology Criteria for Adverse Events, version 4.0 of the National Cancer Institute. Moreover, safety laboratory, 12-lead ECG, vital sign measurements, and physical examinations were performed.

#### Pharmacokinetics

*C*_max_ and *t*_max_ were determined by direct assessment of the observed concentration versus time curves. The area under the curve until the last quantifiable concentration (AUC_0−t_) was estimated by a linear up/log down method if ≥3 values were available and extrapolated to infinity (AUC_inf_), if the extrapolated part was <30 %. The terminal elimination half-life (*T*_1/2_) was calculated as the ratio of log_e_^2^ to the apparent terminal phase rate constant (*λ*_z_), determined through unweighted linear regression analysis on ≥3 log-transformed concentrations on the linear portion of the terminal slope, excluding the peak concentration. In general, points maximizing *R* ^2^ up to at least 0.9 were included for linear regression.

#### Pharmacodynamics

cIAP-1 levels were measured in tumor tissue and surrogate tissue as available. Peripheral blood mononuclear cells (PBMCs) were analyzed for DEBIO1143-induced cIAP-1 degradation. Plasma native cytokeratin-18 (M65) or caspase-3 generated cytokeratin-18 fragments (M30), interleukin 8 (IL8), chemokine ligand 2 (CCL2, MCP1), and tumor necrosis factor α (TNFα) were measured by ELISA as markers of epithelial cell death and inflammation on days 1 (predose, 1, 3, 6, and 12 h postdose), 2, and 5.

#### Efficacy

Tumor evaluations were scheduled before therapy and after every other cycle of therapy. Changes were determined by physical examination, tumor markers, or standard imaging techniques. Response to DEBIO1143 was assessed for solid tumors based on RECIST guidelines, version 1.1 [10] and for lymphoma as per the Revised Response Criteria for Malignant Lymphoma [11]. Read-outs were complete response (CR), partial response (PR), stable disease (SD), and disease progression (DP).

### PK and PD analyses

DEBIO1143 plasma concentrations were measured using a validated LC–MS/MS assay. For the detection of cIAP-1 in paraffin-embedded human tissue, a validated immunohistochemical (IHC) assay was used. DEBIO1143-induced cIAP-1 degradation was measured in PBMCs using Western blot. Plasma biomarkers were measured through commercial ELISAs. All laboratory analyses were performed by MPI Research, Inc. (PK) and Mosaic Laboratory, LLC (PD).

### Data analysis

Descriptive statistics were presented as appropriate. Safety laboratory assessments, vital signs, body weight, ECOG, ECG, as well as PD and tumor response data were compared over time to assess change from baseline during treatment and follow-up. In case of sufficient sample size, Wilcoxon matched pairs test was used for inferential comparisons. Data were analyzed using the Statistical Analysis System (SAS), version 9.1.3. Individual PK parameters were derived using WinNonlin (version 5.3, Pharsight Corp., Mountain View, CA).

## Results

**Fig. 1:**
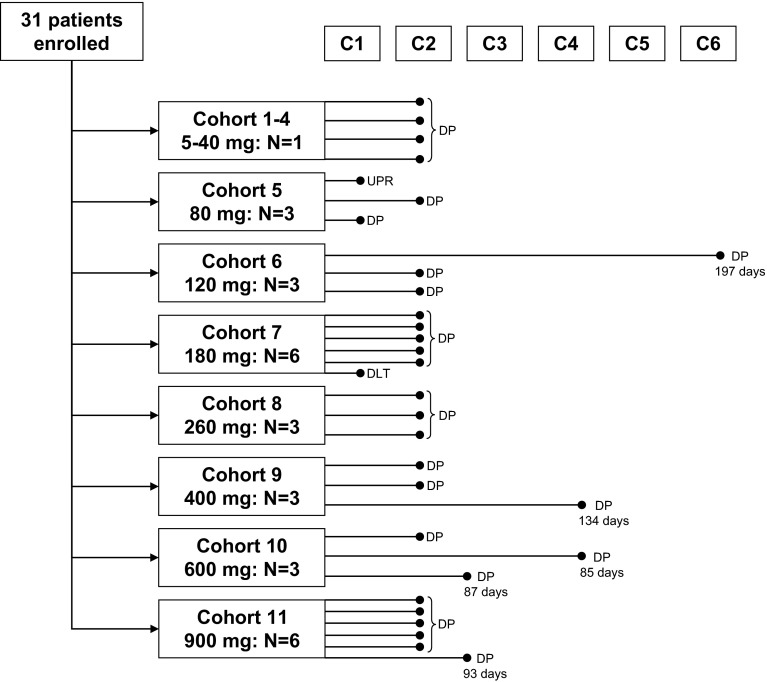
Patient flowchart according to CONSORT. *DP* disease progression (*second line*: duration of stable disease); *UPR* upon patient request

From March 30, 2010, until August 8, 2012, 31 patients, 12 men (38.7 %) and 19 women (61.3 %) with a mean age of 52 ± 11 years, were enrolled and treated in 11 cohorts with oral DEBIO1143 doses ranging from 5 to 900 mg (5, 10, 20, 40, 80, 120, 180, 260, 400, 600, 900 mg; Fig. 1). The great majority of patients were Caucasians (87.1 %; one American Indian, one Asian, and two African-American) with metastatic solid tumors except one lymphoma patient. Most common were colorectal cancer in 14 patients (45.1 %), melanoma in three (9.7 %), and NSCLC in two (6.5 %); remaining tumor entities (38.7 %) were represented by one patient only (adrenal, angiosarcoma, appendiceal, breast, gastroesophageal, hemangiopericytoma, Hurthle cell, myoepithelial, ovarian, salivary gland, SCC penis).

Patients were treated with DEBIO1143 for up to 117 days and all patients completed at least one cycle; 2 cycles: 27 (87.1 %) patients; 3 and 4 cycles: 5 (16.1 %) patients each; 5 and 6 cycles: 2 (6.5 %) patients each; 7 and 8 cycles: one patient each (3.2 %) (Fig. 1; median 2 cycles). A drug-related grade 2 fatigue in a patient treated with 80 mg prompted expansion to 3-patient cohorts. Subsequently, a grade 3 reversible ALT elevation in a patient receiving 180 mg was the only reported DLT which resulted in the expansion of this cohort to six patients. Dose was escalated to 900 mg daily before enrollment was halted due to the excessive number of pills to be taken. Thus, the MTD was not reached.

### Safety

Of 31 patients in the safety population, 30 (96.8 %) experienced 242 AEs of which 82 (33.9 %) were considered related to study drug (ADRs). Most AEs were of mild to moderate severity and neither incidence nor severity increased with dose. The most affected organ systems were gastrointestinal, general, and skin and subcutaneous disorders (Table 1) with fatigue, nausea, and vomiting as the most common treatment-related AE, each occurring in >10 % of patients (Suppl. 1). A total of eight patients (25.8 %) experienced 13 SAEs (constipation, intestinal obstruction, asthenia, pain, cerebrovascular accident, cranial nerve disorder, urinary retention (once each); nausea, vomiting, dyspnoea (twice each)), none of which was considered related to study drug. No patient died during the study. Four (12.9 %) patients discontinued drug treatment due to AEs (ALT increase, cranial nerve disorder, abdominal pain, dyspnoea), of which only the ALT increase was considered related to study drug. This DLT was a fivefold, but asymptomatic ALT increase along with grade 2 elevations of other liver function tests after the first treatment cycle in a 57-year-old white female patient with metastatic colon cancer. ALT but not the other liver function tests had considerably decreased 30 days posttreatment although metastatic disease in the liver may have been a contributing factor. ALT, AST, and GGT were within normal ranges in all remaining patients.

**Table 1:** Number of patients with ADRs and ADR frequency by system organ class

Dose cohort	≤40 mg	80 mg	120 mg	180 mg	260 mg	400 mg	600 mg	900 mg	Total	%
*N*	4	3	3	6	3	3	3	6	31	100
Gastrointestinal disorders	1	2	1	2	1	1	2	3	13	41.9
General disorders and administration site reactions	1	2		3		1	2	1	10	32.3
Skin and subcutaneous disorders		1	1	2		1	1	3	9	29.0
Metabolism and nutrition disorders	1			1		1	1	2	6	19.4
Musculoskeletal and connective tissue disorders	1	1				1	1		4	12.9
Nervous system disorders				1		1		1	3	9.7
Investigations				3					3	9.7
Respiratory, thorax, and mediastinum disorders	1						1		2	6.5
Eye disorders				1					1	3.2
Psychiatric disorders		1							1	3.2
Vascular disorders	1								1	3.2
Patients with any related AE	1	2	1	5	1	1	3	4	18	58.1

No clinically meaningful trends were seen in measurements of safety laboratory, ECG, vital signs, body weight, or ECOG performance status. There were no dose reductions, delays, or modifications due to AEs or lack of tolerability.

### Pharmacokinetics

**Fig. 2:**
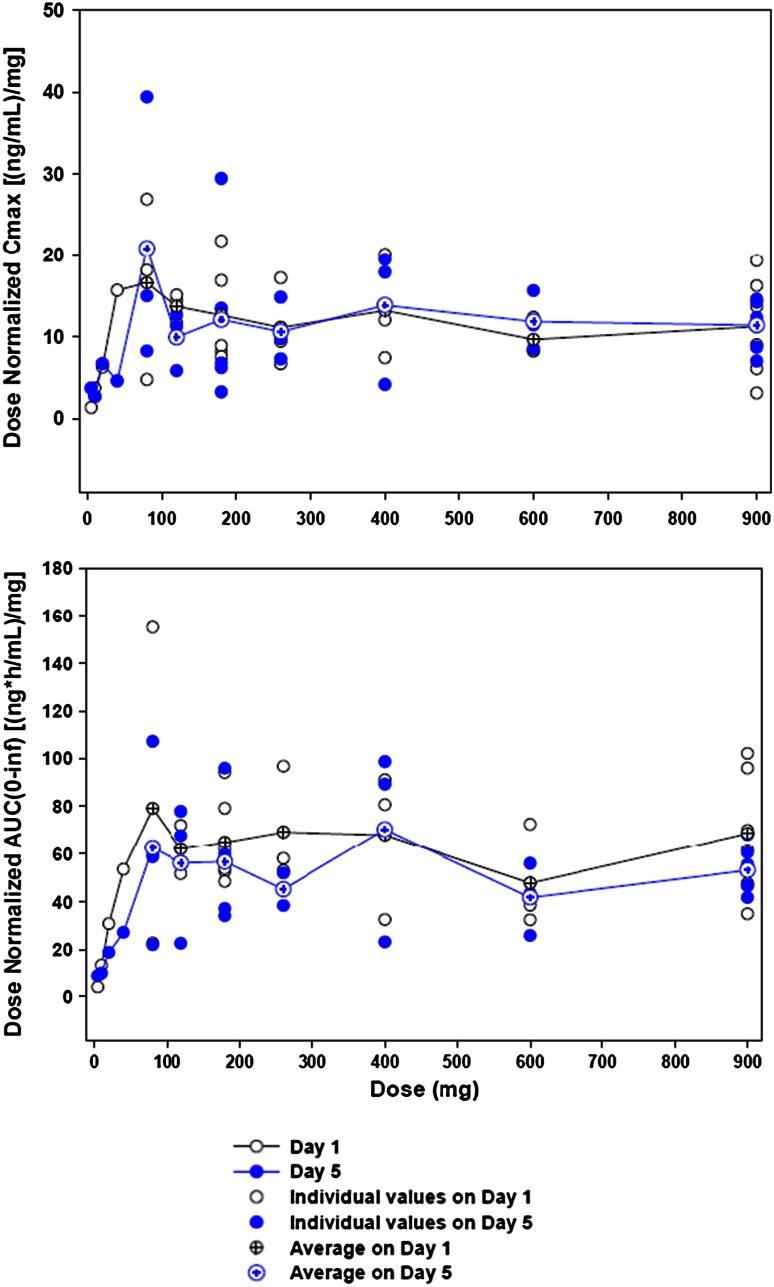
Dose proportionality of *C*_max_ and AUC_inf_

DEBIO1143 drug levels at doses of ≥80 mg exceeded levels that have demonstrated activity in animal models. DEBIO1143 showed rapid absorption after oral intake with peak plasma concentrations within 1–3 h. Overall, increases in *C*_max_ and AUC were proportional to doses ≥80 mg (Fig. 2). In general, PK disposition varied among individuals (Table 2), but mean *T*_1/2_ of DEBIO1143 on day 1 was consistent [5.2–7.1 h] regardless of the dose. No evidence of drug accumulation was observed over the 5-day dosing period.

**Table 2:** Pharmacokinetics of DEBIO1143, means (standard deviation)

Dose (mg)	*n*	*C*_max_ (ng/mL)		*T*_max_** (h)		AUC_0−t_ (ng*h/mL)		AUC_0−inf_ (ng*h/mL)		*T*_1/2_ (h)		Day 1:5 ratio
		Day 1	Day 5	Day 1	Day 5	Day 1	Day 5	Day 1	Day 5	Day 1	Day 5	
5	1	6.69	18.5	3.0	1.6	19.6	43.4	ND	ND	ND	ND	ND
10	1	37.4	26.9	1.0	3.5	129	103	152	117	2.46	2.53	0.77
20	1	126	134	1.0	0.5	615	375	643	451	5.44	3.37	0.70
40	1	627	184	1.0	6.1	2140	1090	2300	ND	6.38	ND	ND
80	3	1330 (891)	1670 (1310)	2.0 (0.7–3.0)	2.0 (0.6–2.0)	6390 (5420)	5020 (3450)	6660 (5700)	5920 (4270)	5.22 (0.890)	2.69 (0.593)	0.96 (0.13)
120	3	1650 (217)	1190 (432)	1.0 (0.5–1.0)	2.0 (1.0–3.0)	6890 (1010)	5430 (2570)	7460 (1230)	6770 (3460)	6.39 (1.04)	2.61 (0.505)	0.88 (0.36)
180	7	2130 (1010)	2160 (1550)	2.0 (0.5–6.0)	2.0 (0.5–6.0)	10,600 (2990)	7210 (3620)	11,400 (3080)	9980 (5150)	6.06 (1.05)	4.14 (1.72)	0.90 (0.45)
260	3	2890 (1420)	2760 (999)	3.0 (1.0–3.0)	2.0 (1.1–6.0)	16,300 (5710)	10600 (2910)	17,900 (6260)	11,700 (2520)	6.59 (0.416)	3.29 (0.573)	0.82 (0.14)
400	3	5280 (2570)	5540 (3370)	1.0 (0.6–2.0)	0.5 (0.5–2.0)	25,000 (11,300)	20,100 (11,700)	27,000 (12,400)	26,500 (15,200)	6.27 (0.367)	3.70 (0.390)	0.92 (0.19)
600	3	5780 (1410)	7110 (2140)	1.1 (0.6–1.1)	1.0 (0.5–1.1)	27,000 (12,500)	20,200 (7530)	28,700 (13,400)	25,000 (9090)	5.75 (0.478)	3.54 (1.67)	0.90 (0.21)
900	6	10,100 (5600)	10,300 (2710)	1.5 (1.0–3.0)	1.5 (1.0–3.0)	56,100 (21,900)	36,900 (8210)	61,500 (23,500)	47,000 (8550)	7.11 (0.675)	3.15 (0.408)	0.83 (0.25)

*ND* not determined

** Median (minimum–maximum)

### Pharmacodynamics

#### cIAP1 levels in tissues and PBMCs

**Fig. 3:**
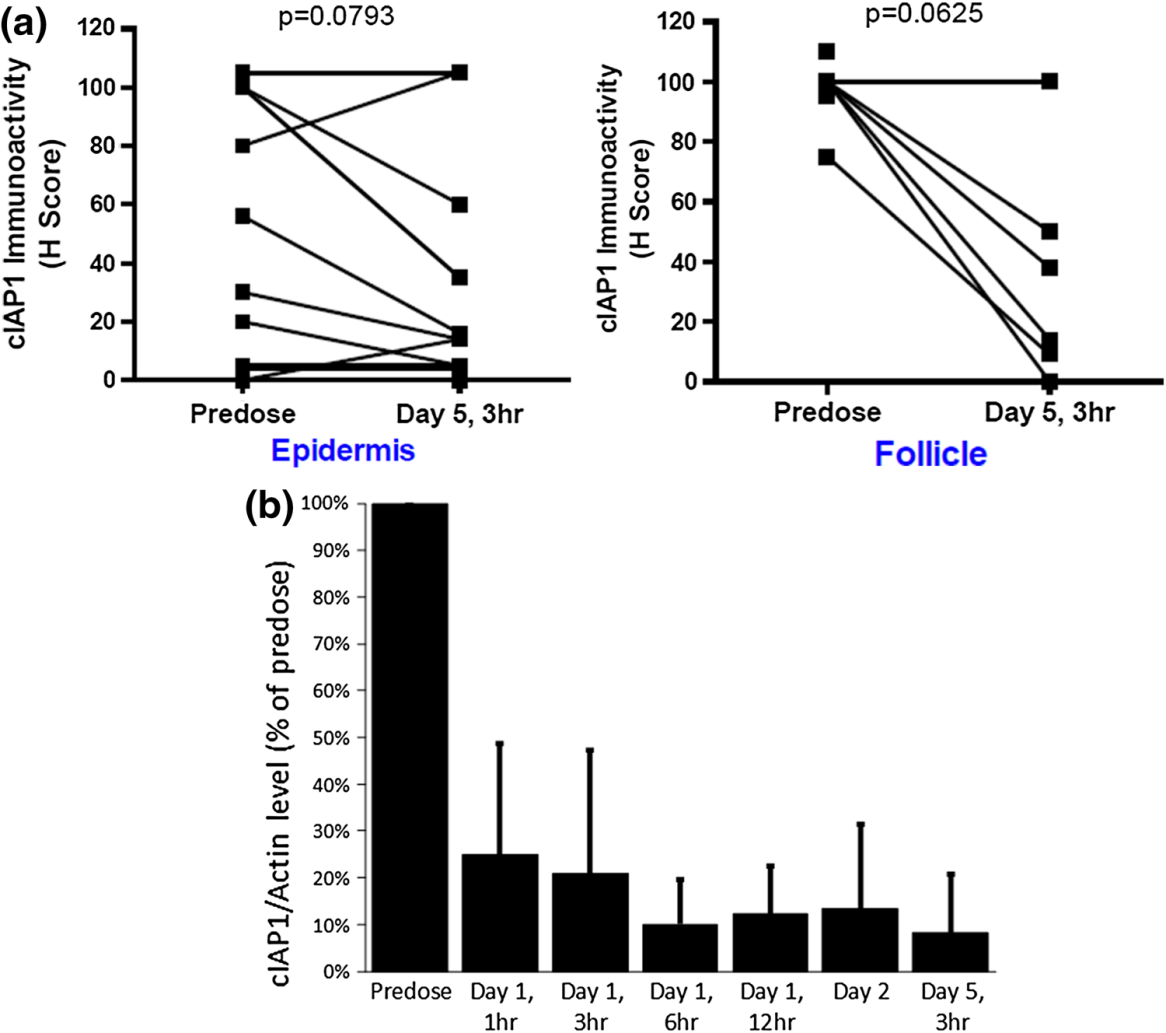
Expression of cIAP. **a** in skin biopsies of 12 patients (H-scores; on the *top*). **b** in PBMC (quantitative Western blot results as % from baseline) across doses (on the *bottom*; for results per dose see Suppl. 2)

A rapid and substantial cIAP1 degradation was observed in tumor or surrogate tissues. IHC staining of cIAP1 in skin biopsies of 12 patients revealed a trend for a decrease in the level of cIAP1 (Fig. 3a). In baseline and on-treatment tumor biopsies from two patients with melanoma, cIAP1 was detected with intensities ranging from 0 to 2+. In the patient treated with DEBIO1143 at 120 mg/day, the immunoactivity of cIAP1 decreased from 150 (predose) to 130 on day 5. By contrast, only negligible effect on the percentage of cIAP1-positive cells was observed in the tumor biopsies of the other melanoma patient treated at 400 mg/d.

The expression of cIAP1 was evaluable in PBMCs from 28 patients with doses above 80 mg using Western blot (Fig. 3b; Suppl. 2). In 20 patients, cIAP1 was readily detectable at baseline but undetectable or extremely low in eight patients. In all patients with detectable cIAP1, DEBIO1143 led to rapid and persistent cIAP1 degradation regardless of dose.

#### Plasma levels of TNFα, IL8, CCL2, and M30/M65

**Fig. 4:**
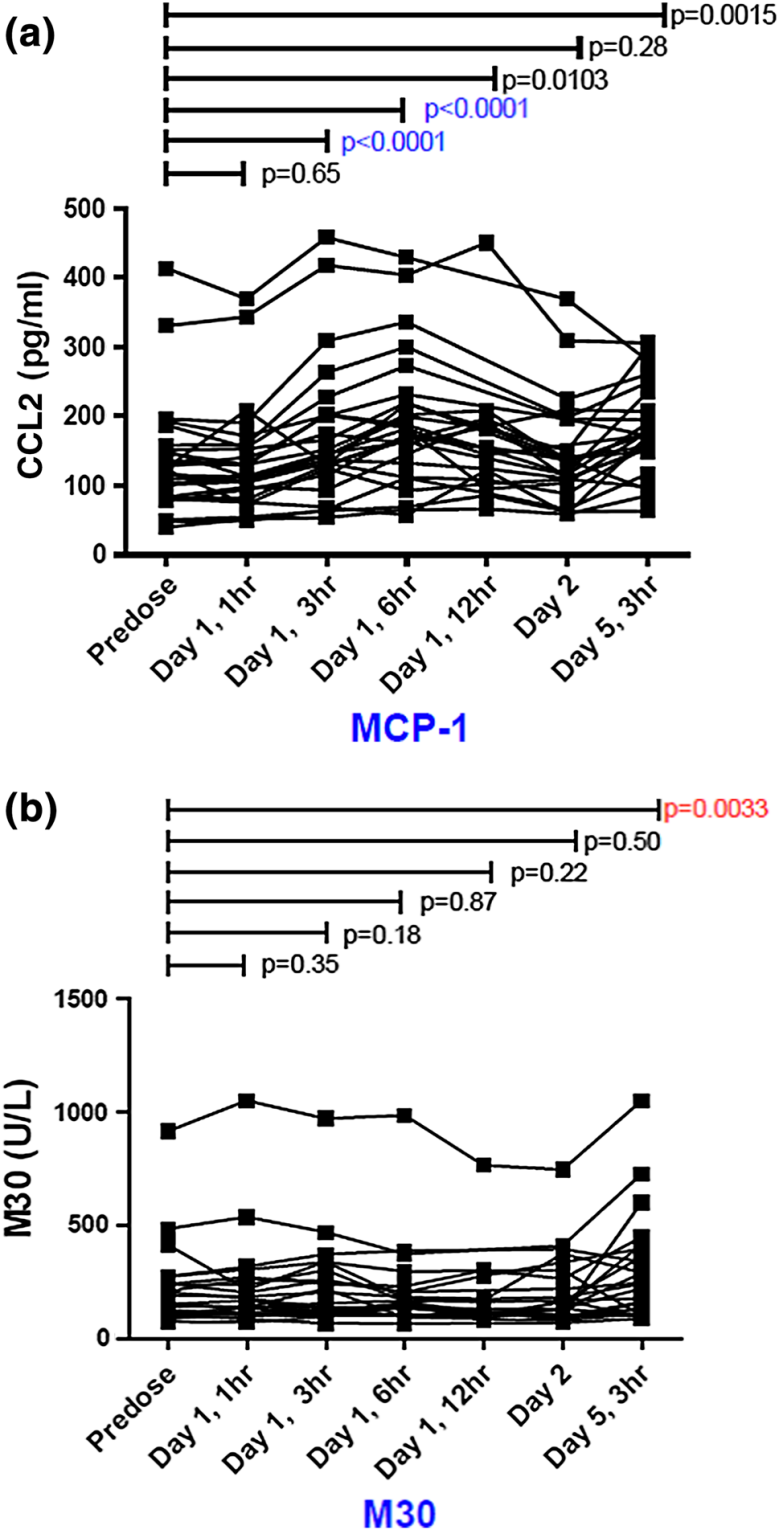
Pharmacodynamic measurements

In total, 173 plasma samples from 25 patients were measured for TNFa, CCL2, and IL8, biomarkers mechanistically related to DEBIO1143. In 108 samples, TNFα was below the limit of detection; four out of five patients with detectable TNFα levels showed some increase postdose. CCL2 was detectable in 25 patients and increased in 14 patients across all dose levels; however, CCL2 increased in five out of six patients dosed with 900 mg (mean increase 45 %, range −6 to 54 %). Increases became significant 3 and 6 h postdose (*p* < 0.0001, Fig. 4). By contrast, neither an increase in plasma IL8 nor any correlation with DEBIO1143 exposure was observed. Plasma level of M30 increased significantly on day 5 (*p* < 0.0033; Fig. 4). However, no significant changes were found for M65 or for the M30:M65 ratio. None of the biomarkers showed any apparent relationship to the treatment response.

### Antitumor activity

No patient had a complete or partial response. One patient with metastatic melanoma with latero-cervical lymph node involvement showed an 11 % reduction in target lesion dimensions at 400 mg/day. Progression in the same nodes was noted after six cycles. Stable disease as best response was seen in five patients (16.1 %) for a median duration of 93 days (range 85–197 days; Fig. 1). All these patients had different cancer types (Hurthle cell, melanoma, breast, rectal, hemangiopericytoma).

## Discussion

Several SMAC mimetic IAP antagonists have entered clinical development, including DEBIO1143 (formerly AT-406), HGS1029 (formerly AEG-40826), GDC-0917 and -0152, LCL-161, and birinapant (TL-32711). The latter two compounds have entered phase II trials. Our results on DEBIO1143 add to the existing body of evidence from these clinical trials on SMAC mimetic IAP antagonists. In general, tolerability and safety of this class of drugs have been acceptable [12–14]. These trials have not revealed any consistent AE in humans [3, 12–14] except an increased incidence of Bell’s Palsy syndrome at higher doses of birinapant which can be prevented by dose titration in the initial treatment cycle [15]. In this regard, it is noteworthy that the cerebrovascular accident and cranial nerve disorder in our study were both unlikely related to DEBIO1143. The former occurred 23 days after last intake, and signs of thrombocytopenia, coagulopathy, or hypertension were absent during the two cycles of treatment as well as at the time of the event. By contrast, an occult metastasis could not be ruled out. The cranial nerve disorder was not Bell’s Palsy syndrome as it affected cranial nerve 5 rather than 7; it was most consistent with progression of leptomeningeal disease. In general, there was no grade 4–5 treatment-related AE at all, even during treatment for over 6 months. The only case of transient G3 hepatotoxicity is consistent with preclinical data and linked to the mechanism of action of SMAC mimetic IAP inhibitors [16, 17].

Our PK data in humans confirmed drug exposures at or above those needed for activity in preclinical models [7]. PK disposition of DEBIO1143 is suitable for once daily dosing over 5 consecutive days every 3 weeks. DEBIO1143 showed proportional plasma increases at doses >80 mg in our study (Fig. 2). However, beyond this threshold, neither PD nor antitumor activity showed any dose relationship, but all doses resulted in the degradation of cIAP-1 in PBMCs and at least in a trend for a decrease in cIAP levels in surrogate skin tissue. The on-target activity of DEBIO1143 was also supported by the significant increase in CCL2 plasma levels (Fig. 4), which might be a consequence of the cIAP-1 degradation through modulation of NF-κB. CCL2, actually a marker of inflammation, has also been associated with the stimulation of a host antitumor response [18] which would be in line with the observed M30 increase indicating drug-induced epithelial apoptosis [19].

However, interpretation of PD data remains limited due to the small samples sizes of dose groups and the lacking dose–response relationship. Our data also remain inconclusive regarding a recommendable dose, although 900 mg/day resulted in acceptable tolerability and in exposures with proven activity in preclinical experiments. It may thus serve at least as a starting dose for phase II. In addition, the modest clinical activity of IAP inhibitors shown in unselected refractory cancer patients so far suggests the need for combination approaches and screening for more sensitive subpopulations.

## Electronic supplementary material

Below is the link to the electronic supplementary material.

Supplementary material 1 (DOC 195 kb)
