# Supplementary material for: Safety, pharmacokinetics, and pharmacodynamic properties of oral DEBIO1143 (AT-406) in patients with advanced cancer: results of a first-in-man study
Source: Cancer Chemother Pharmacol. 2015 Feb 27;75(4):851–9. doi: 10.1007/s00280-015-2709-8 (PMC4365270; doi:10.1007/s00280-015-2709-8)
Supplement: Supplementary file 1 — Supplementary material 1 (DOC 195 kb) [file 280_2015_2709_MOESM1_ESM.doc]

# Supplementary data

**Suppl. 1 ADRs by System Organ Class and Preferred Term**

| **Cohort (Dose mg)** | **1-4 (≤40)** | | **5 (80)** | | **6 (120)** | | **7 (180)** | | **8 (260)** | | **9 (400)** | | **10 (600)** | | **11 (900)** | | **Total AE** | | **Total pat** | |
| --- | --- | --- | --- | --- | --- | --- | --- | --- | --- | --- | --- | --- | --- | --- | --- | --- | --- | --- | --- | --- |
| **N** | **4** | | **3** | | **3** | | **6** | | **3** | | **3** | | **3** | | **6** | | **242 (100%)** | | **31 (100%)** | |
| **System Organ Class** / • PT | AE | pat | AE | pat | AE | pat | AE | pat | AE | pat | AE | pat | AE | pat | AE | pat | AE | % | pat | % |
| **Eye** |  |  |  |  |  |  | **1** | **1** |  |  |  |  |  |  |  |  | **1** | **0.4** | **1** | **3.2** |
| - Blepharospasm |  |  |  |  |  |  | 1 | 1 |  |  |  |  |  |  |  |  | 1 | 0.4 | 1 | 3.2 |
| **Gastrointestinal** | **1** | **1** | **6** | **2** | **2** | **1** | **8** | **2** | **2** | **1** | **6** | **1** | **2** | **2** | **3** | **3** | **30** | **12.4** | **13** | **41.9** |
| - Abdominal Distension |  |  |  |  |  |  |  |  |  |  | 1 | 1 |  |  | 1 | 1 | 2 | 0.8 | 2 | 6.5 |
| - Constipation |  |  | 1 | 1 |  |  |  |  | 1 | 1 | 1 | 1 |  |  |  |  | 3 | 1.2 | 3 | 9.7 |
| - Diarrhoea |  |  |  |  |  |  | 1 | 1 |  |  | 1 | 1 |  |  | 1 | 1 | 3 | 1.2 | 3 | 9.7 |
| - Dry Mouth | 1 | 1 |  |  |  |  | 1 | 1 |  |  |  |  |  |  |  |  | 2 | 0.8 | 2 | 6.5 |
| - Dyspepsia |  |  | 2 | 1 |  |  |  |  |  |  |  |  |  |  |  |  | 2 | 0.8 | 1 | 3.2 |
| - Nausea |  |  | 3 | 2 |  |  | 3 | 2 |  |  | 2 | 1 | 1 | 1 | 1 | 1 | 10 | 4.1 | 7 | 22.6 |
| - Stomatitis |  |  |  |  |  |  | 1 | 1 | 1 | 1 |  |  |  |  |  |  | 2 | 0.8 | 2 | 6.5 |
| - Vomiting |  |  |  |  | 2 | 1 | 2 | 1 |  |  | 1 | 1 | 1 | 1 |  |  | 6 | 2.5 | 4 | 12.9 |
| **General & admin. site** | **1** | **1** | **3** | **2** |  |  | **4** | **3** |  |  | **2** | **1** | **2** | **2** | **1** | **1** | **13** | **5.4** | **10** | **32.3** |
| - Fatigue |  |  | 3 | 2 |  |  | 4 | 3 |  |  | 1 | 1 | 2 | 2 |  |  | 10 | 4.1 | 8 | 25.8 |
| - Irritability | 1 | 1 |  |  |  |  |  |  |  |  |  |  |  |  |  |  | 1 | 0.4 | 1 | 3.2 |
| - Malaise |  |  |  |  |  |  |  |  |  |  | 1 | 1 |  |  |  |  | 1 | 0.4 | 1 | 3.2 |
| - Oedema Peripheral |  |  |  |  |  |  |  |  |  |  |  |  |  |  | 1 | 1 | 1 | 0.4 | 1 | 3.2 |
| **Investigations** |  |  |  |  |  |  | **4** | **3** |  |  |  |  |  |  |  |  | **4** | **1.7** | **3** | **9.7** |
| - ALT Increased |  |  |  |  |  |  | 2 | 1 |  |  |  |  |  |  |  |  | 2 | 0.8 | 1 | 3.2 |
| - Weight Decreased |  |  |  |  |  |  | 2 | 2 |  |  |  |  |  |  |  |  | 2 | 0.8 | 2 | 6.5 |
| **Metabolism & Nutrition** | **1** | **1** |  |  |  |  | **2** | **1** |  |  | **1** | **1** | **1** | **1** | **3** | **2** | **8** | **3.3** | **6** | **19.4** |
| - Decreased Appetite |  |  |  |  |  |  | 2 | 1 |  |  | 1 | 1 | 1 | 1 |  |  | 4 | 1.7 | 3 | 9.7 |
| - Dehydration | 1 | 1 |  |  |  |  |  |  |  |  |  |  |  |  |  |  | 1 | 0.4 | 1 | 3.2 |
| - Hyperglycaemia |  |  |  |  |  |  |  |  |  |  |  |  |  |  | 1 | 1 | 1 | 0.4 | 1 | 3.2 |
| - Hypocalcaemia |  |  |  |  |  |  |  |  |  |  |  |  |  |  | 1 | 1 | 1 | 0.4 | 1 | 3.2 |
| - Hyponatraemia |  |  |  |  |  |  |  |  |  |  |  |  |  |  | 1 | 1 | 1 | 0.4 | 1 | 3.2 |
| **Musculoskeletal & Connective Tissue** | **1** | **1** | **1** | **1** |  |  |  |  |  |  | **2** | **1** | **1** | **1** |  |  | **5** | **2.1** | **4** | **12.9** |
| - Muscle Spasms |  |  | 1 | 1 |  |  |  |  |  |  | 1 | 1 |  |  |  |  | 2 | 0.8 | 2 | 6.5 |
| - Musculoskeletal Stiffness |  |  |  |  |  |  |  |  |  |  | 1 | 1 |  |  |  |  | 1 | 0.4 | 1 | 3.2 |
| - Myalgia |  |  |  |  |  |  |  |  |  |  |  |  | 1 | 1 |  |  | 1 | 0.4 | 1 | 3.2 |
| - Pain In Extremity | 1 | 1 |  |  |  |  |  |  |  |  |  |  |  |  |  |  | 1 | 0.4 | 1 | 3.2 |
| **Nervous System** |  |  |  |  |  |  | **1** | **1** |  |  | **1** | **1** |  |  | **2** | **1** | **4** | **1.7** | **3** | **9.7** |
| - Headache |  |  |  |  |  |  |  |  |  |  | 1 | 1 |  |  |  |  | 1 | 0.4 | 1 | 3.2 |
| - Memory Impairment |  |  |  |  |  |  | 1 | 1 |  |  |  |  |  |  |  |  | 1 | 0.4 | 1 | 3.2 |
| - Tension Headache |  |  |  |  |  |  |  |  |  |  |  |  |  |  | 2 | 1 | 2 | 0.8 | 1 | 3.2 |
| **Psychiatric Disorders** |  |  | **1** | **1** |  |  |  |  |  |  |  |  |  |  |  |  | **1** | **0.4** | **1** | **3.2** |
| - Nightmare |  |  | 1 | 1 |  |  |  |  |  |  |  |  |  |  |  |  | 1 | 0.4 | 1 | 3.2 |
| **Respiratory, Thorax & Mediastinum** | **1** | **1** |  |  |  |  |  |  |  |  |  |  | **1** | **1** |  |  | **2** | **0.8** | **2** | **6.5** |
| - Dyspnoea |  |  |  |  |  |  |  |  |  |  |  |  | 1 | 1 |  |  | 1 | 0.4 | 1 | 3.2 |
| - Hiccups | 1 | 1 |  |  |  |  |  |  |  |  |  |  |  |  |  |  | 1 | 0.4 | 1 | 3.2 |
| **Skin & subcutaneous** |  |  | **1** | **1** | **1** | **1** | **2** | **2** |  |  | **4** | **1** | **1** | **1** | **4** | **3** | **13** | **5.4** | **9** | **29.0** |
| - Nail Discolouration |  |  |  |  | 1 | 1 | 1 | 1 |  |  |  |  |  |  |  |  | 2 | 0.8 | 2 | 6.5 |
| - Night Sweats |  |  | 1 | 1 |  |  |  |  |  |  | 1 | 1 |  |  | 1 | 1 | 3 | 1.2 | 3 | 9.7 |
| - Pruritus |  |  |  |  |  |  | 1 | 1 |  |  | 1 | 1 |  |  | 1 | 1 | 3 | 1.2 | 3 | 9.7 |
| - Rash |  |  |  |  |  |  |  |  |  |  |  |  | 1 | 1 | 2 | 2 | 3 | 1.2 | 3 | 9.7 |
| - Rash Erythematous |  |  |  |  |  |  |  |  |  |  | 2 | 1 |  |  |  |  | 2 | 0.8 | 1 | 3.2 |
| **Vascular** | **1** | **1** |  |  |  |  |  |  |  |  |  |  |  |  |  |  | **1** | **0.4** | **1** | **3.2** |
| - Hot Flush | 1 | 1 |  |  |  |  |  |  |  |  |  |  |  |  |  |  | 1 | 0.4 | 1 | 3.2 |
| **Any related AE** | **6** | **1** | **12** | **2** | **3** | **1** | **22** | **5** | **2** | **1** | **16** | **1** | **8** | **3** | **13** | **4** | **82** | **33.9** | **17** | **54.8** |

pat: patients

Suppl. 2 Quantitative Western blot results in PBMC by dose: cIAP1/Actin in [% from baseline]

|  | **Dose [mg]** | **120** | **180** | **260** | **400** | **600** | **900** |
| --- | --- | --- | --- | --- | --- | --- | --- |
| ***Pre-dose*** | N | 1 | 7 | 3 | 3 | 3 | 5 |
|  |  | 100% | 100% | 100% | 100% | 100% | 100% |
| ***1h*** | N | 1 | 6 | 3 | 1 | 3 | 5 |
|  | Mean (SD) | 2% (0) | 34% (31) | 39% (33) | 14% (0) | 27% (9) | 11% (8) |
|  | Median (range) | 2 (2-2) | 33 (0-78) | 44 (4-70) | 14 (14-14) | 25 (20-38) | 9 (2-24) |
| ***3h*** | N | 1 | 6 | 3 | 2 | 3 | 4 |
|  | Mean (SD) | 2% (0) | 20% (25) | 45% (50) | 4% (2) | 24% (11) | 15% (23) |
|  | Median (range) | 2 (2-2) | 12 (3-68) | 24 (9-103) | 4 (3-6) | 18 (17-36) | 3 (3-49) |
| ***6h*** | N | 1 | 7 | 3 | 2 | 3 | 4 |
|  | Mean (SD) | 4% (0) | 6% (8) | 9% (4) | 6% (4) | 20% (11) | 14% (13) |
|  | Median (range) | 4 (4-4) | 5 (0-24) | 8 (5-13) | 6 (2-9) | 17 (11-33) | 11 (1-32) |
| ***12h*** | N | 1 | 3 | 2 | n/a | 1 | 2 |
|  | Mean (SD) | 3% (0) | 8% (7) | 14% (5) | n/a | 36% (0) | 9% (2) |
|  | Median (range) | 3 (3-3) | 5 (4-16) | 14 (10-17) | n/a | 36 (36-36) | 9 (7-10) |
| ***24h*** | N | 1 | 6 | 3 | 2 | 3 | 4 |
|  | Mean (SD) | 4% (0) | 9% (10) | 9% (7) | 19% (26) | 29% (36) | 11% (18) |
|  | Median (range) | 4 (4-4) | 5 (3-29) | 6 (5-16) | 19 (0-37) | 14 (3-70) | 3 (0-38) |
| ***99h*** | N | 1 | 6 | 3 | 2 | 2 | 4 |
|  | Mean (SD) | 5% (0) | 2% (2) | 6% (3) | 28% (31) | 14% (19) | 7% (6) |
|  | Median (range) | 5 (5-5) | 0 (0-5) | 6 (3-8) | 28 (6-51) | 14 (0-28) | 8 (0-14) |
